# The Specific Role of Reactive Astrocytes in Stroke

**DOI:** 10.3389/fncel.2022.850866

**Published:** 2022-03-07

**Authors:** Leiyang Li, Jinpeng Zhou, Liying Han, Xun Wu, Yingwu Shi, Wenxing Cui, Shenghao Zhang, Qing Hu, Jin Wang, Hao Bai, Haixiao Liu, Wei Guo, Dayun Feng, Yan Qu

**Affiliations:** Department of Neurosurgery, Tangdu Hospital, Fourth Military Medical University, Xi’an, China

**Keywords:** stroke, reactive astrocyte, polarization, blood brain barrier (BBB), neuro-inflammation

## Abstract

Astrocytes are essential in maintaining normal brain functions such as blood brain barrier (BBB) homeostasis and synapse formation as the most abundant cell type in the central nervous system (CNS). After the stroke, astrocytes are known as reactive astrocytes (RAs) because they are stimulated by various damage-associated molecular patterns (DAMPs) and cytokines, resulting in significant changes in their reactivity, gene expression, and functional characteristics. RAs perform multiple functions after stroke. The inflammatory response of RAs may aggravate neuro-inflammation and release toxic factors to exert neurological damage. However, RAs also reduce excitotoxicity and release neurotrophies to promote neuroprotection. Furthermore, RAs contribute to angiogenesis and axonal remodeling to promote neurological recovery. Therefore, RAs’ biphasic roles and mechanisms make them an effective target for functional recovery after the stroke. In this review, we summarized the dynamic functional changes and internal molecular mechanisms of RAs, as well as their therapeutic potential and strategies, in order to comprehensively understand the role of RAs in the outcome of stroke disease and provide a new direction for the clinical treatment of stroke.

## Introduction

Stroke is an acute cerebrovascular disease in which the blood cannot supply the brain generally due to the sudden rupture or blockage of the cerebrovascular. In 2019, stroke resulted in the death of 6.55 million people worldwide, accounting for 11.6% of the total number of deaths. About 3/4 of stroke survivors have neurological disorders of varying degrees, and 15–30% of patients are permanently disabled (Lai et al., 2002; Wu et al., 2019; GBD 2019 Stroke Collaborators, 2021). Therefore, stroke has been the second cause of death and the leading cause of severe disability globally. After the stroke, damage factors such as blood components infiltrating into the brain parenchyma, necrotic cell debris, and reactive oxygen species (ROS) often lead to neuronal degeneration or apoptosis, increasing permeability of BBB, inflammatory response, and brain edema (Iadecola et al., 2020). This is likewise the main reason for the high disability rate of stroke patients. Thus, finding effective intervention methods to control secondary brain injury (SBI) has become a vital goal to improve the prognosis of stroke patients (Chamorro et al., 2016; Lambertsen et al., 2019).

After the stroke, SBI involves vascular endothelial cells, neurons, glial cells, and other cell types, which interact to jointly determine the damage response’s occurrence, development, and outcome. Astrocytes are the most abundant subtype among these cells, highly expressing the interleukin-1 receptor (IL-1R), tumor necrosis factor receptor (TNFR), interferon-γ receptor (IFN-γR), and other inflammatory signal receptors (Lovatt et al., 2007; Wang et al., 2019), so they are one of the cells targeted for inflammatory factors. Moreover, astrocytes can also secrete CXCL12, CCl2, C1q, and other inflammatory factors to aggravate SBI (Yamada et al., 1992). Therefore, astrocytes are likely to be important targets for regulating SBI. But what are the possible roles and mechanisms of astrocytes in SBI?

Accumulating evidence supports that astrocyte undergoes significant changes after the stroke in morphology, gene expression, and cell proliferation, and these cells are known as RAs (Zisis et al., 2021). Currently, it is known that the RAs’ effects may include scarring, neurotrophic factor secretion, BBB damaging and repairing, and inhibition of synaptic formation. However, the formation and mechanisms of RAs have yet to be clearly understood. This review focuses mainly on the morphological and phenotypic changes of astrocytes after the stroke and analyses the molecular biological mechanisms behind them, reveals the pathologic mode of RAs, summarizes the application of new techniques in RAs, and outlines the therapeutic potential of astrocytes in stroke. We expect to find a comprehensive treatment strategy to reduce stroke SBI and support patient recovery based on an in-depth understanding of RAs.

## Reactive Astrocytes

After the stroke, astrocytes express multiple Toll-like receptors (TLR), including TLR4, TLR2, and TLR3, which respond to distress-associated molecular patterns (DAMPs) released by injured cells (Sofroniew, 2014; Wang et al., 2019). Besides, neurotransmitters released by damaged neurons and pro-inflammatory factors secreted by adjacent cells act together on astrocytes, leading to significant changes in astrocyte morphology, molecular expression, and other levels (Johnston, 2005; Lan et al., 2017). To distinguish them from resting astrocytes, the drastically altered astrocytes are named RAs.

### Morphological Changes of Reactive Astrocytes

Morphological changes of RAs are the most observable changes in stroke. A typical mature astrocyte has an oval body and several main branches, each divided into secondary and tertiary branches, which in turn are divided into fine processes. Each astrocyte occupies a separate, non-overlapping region in the adult brain, where astrocyte processes interact with synapses to form a delicate and complex network of processes. However, the relatively independent relationship between processes was broken after the stroke. Huang et al. (2014) found that hypertrophic processes of reactive astrocytes were overlapping and crisscrossed in the surrounding area of ischemic infarction, forming a capsule layer to cover the Infarction area. The morphological changes of RAs are spatially different. The diameter, length, and branching levels of RAs processes in the ischemic penumbra were higher than those in the contralateral hemisphere and distal astrocytes far from the ischemic core. Furthermore, the volume and branching structure of processes increased with decreasing distance from the lesion (Wagner et al., 2013). The morphological changes of RAs were likewise time-dynamic. Li induced photothrombosis-induced ischemia in mice and found that the expression level of glial fibrillary acidic protein (GFAP) in RAs was significantly increased with the progressive hypertrophy of processes in the early stage of injury, but the length of RAs did not change markedly. On the sixth day, RAs were densely packed, and their processes form linear structures and extend toward the ischemic core. After 10 days, processes extend further and RAs transition to scar-forming astrocytes, which wrap around the damaged area and separate it from normal tissue (Li et al., 2014). Astrocyte scar formation is an imperative feature of RAs’ morphologic changes after stroke. RAs secret a large amount of chondroitin sulfate proteoglycan (CSPG) and other extracellular matrix components, which together with microglia, oligodendrocytes, and other cellular components form glial scars. The glial scar can wrap around the injury site, limiting further leakage of blood components and monocyte infiltration, thereby inhibiting progressive enlargement of the ischemic penumbra (Yang, 2020). The duration of the astrocyte scar is related to the severity of the injury. Mild astrocyte activation may subside over time. In more severe cases, RAs form permanent glial scars, which inhibit axonal regeneration and are associated with poor neurological recovery after the stroke (Buss et al., 2004; Escartin et al., 2019).

Changes in the main processes of RAs during stroke have been carried out relatively detailed. Whereas limited by cell imaging technology, there is still a lack of relevant studies on the ultrastructural morphological changes of RAs endfeet and adjacent cells such as neurons and vascular endothelial cells at all stages of stroke. It is up to later researchers to complete these researches and fill in these gaps.

### Marker of Reactive Astrocytes

The original definition of RAs was closely related to GFAP (Eng et al., 1971). GFAP is one of the major components of astrocytes’ intermediate filaments. GFAP mRNA and protein expression were significantly up-regulated in astrocytes under various injury factors, so GFAP was considered a universal marker of RAs in initial studies. However, Sun found that GFAP could only stain 15% of individual astrocytes by single-cell dye injection, proving that GFAP is not an absolute marker of astrocytes (Sun and Jakobs, 2012). GFAP was also not strictly correlated with astrocyte reactivity. the expression level of GFAP in the suprachiasmatic nucleus fluctuated with circadian rhythm, suggesting that the changes of GFAP expression may be related to normal physiological functions (Gerics et al., 2006). These indicated that GFAP could not be fully and accurately reflect the morphology and reaction state of RAs, and its role as a general marker of RAs was limited.

Recent transcription studies have identified many astrocytes specific genes. Cahoy used fluorescence-activated cell sorting (FACS) to isolate astrocytes from S100B-EGFP Reporter mice and microarray analysis of over 20,000 genes at different postnatal ages from day 1 to 30. It was found that Aldh1L1 was more widely expressed in astrocytes than GFAP (Cahoy et al., 2008). Previous literature report that astrocytes almost entirely express SOX9 expression in the adult brain and that its expression is not down-regulated in aging astrocytes. Notably, SOX9 expression increases with astrocyte activation in multiple disease models (Sun et al., 2017). Compared with GFAP, these markers are more specific for some types of RAs and have been widely used in the studies of various CNS diseases. Zhang and Lundquist both used SOX9 as a marker of RAs in their studies (Lundquist et al., 2019; Zhang Q. et al., 2021). Götz’s study on brain injury and Michalovicz’s study on the neurotoxicity of RAs also used Aldh1L1 to specifically label RAs (Michalovicz et al., 2019; Götz et al., 2021).

Based on current studies, these markers have shown excellent performance in studying RAs. The development of single-cell sequencing technology also holds out the hope of finding new markers with higher specificity and sensitivity. In addition, we need to explore the change characteristics of different markers in different lesion sites and disease stages and build a comprehensive and efficient RAs marker system to serve for future research on RAs in stroke.

### Phenotypes of Reactive Astrocytes

Previous studies have discovered that RAs activate multiple signaling pathways and have complex molecular expression patterns, with opposite or similar functional states. According to the functional characteristics, RAs can be divided into two types, namely, neuroinflammatory type induced by lipopolysaccharide and neuroprotective type induced by ischemic stroke (Zamanian et al., 2012). This is analogous to microglia changing their phenotypes and functions according to environmental changes (Loane and Kumar, 2016; Bai et al., 2020). Therefore, referring to the M1/M2-like polarization classification of microglia, researchers classified RAs into C3d^+^/GFAP^+^ A1 and S100A10^+^/GFAP^+^ A2 phenotypes (Liddelow and Barres, 2017; Zhang H. et al., 2021). Since then, the study of RAs has been incorporated into the A1/A2 paradigm, and a series of A1/A2 related genes and marker molecules have been proposed (see Table 1). In general, the NF-κB signaling pathway mediates the formation of neuroinflammatory astrocytes, which damage neurons by various ways, such as upregulating complement cascade genes, secreting inflammatory cytokines and saturated fatty acids that induce neuronal apoptosis, and reducing the secretion of GPCG4/6, SPARCL1, and ThBS1/2 with neurotrophic function (Stevens et al., 2007; Liddelow et al., 2017; Guttenplan et al., 2021). In contrast, neuroprotective type astrocytes mediated by JAK2/STAT3 signaling pathway up-regulate many neurotrophic factors and promote neuronal survival and growth (Zhu et al., 2013; Rakers et al., 2019), suggesting that this type of astrocytes may have a “beneficial” repair function.

**TABLE 1:** Type A1/A2 astrocyte specific genes.

Sort	Marker	Known function	Comments	References
Pan	Lcn2	Steroids, lipopolysaccharides, iron, and fatty acids trafficking protein	Lcn2-deficiency showed a significant cognitive decline, white matter damage, blood-brain barrier permeability	Kim et al., 2017; Rakers et al., 2019
	S1pr3	Regulation of angiogenesis and vascular endothelial cell function	Decreased endothelial cell adhesion and increased BBB permeability	Van Doorn et al., 2010; Gril et al., 2018
	Timp1	Metallopeptidase inhibitor	Involved in extracellular matrix maintenance and remodeling	Gardner and Ghorpade, 2003; Houben et al., 2020
	Hspb1	Heat shock protein	Prevents protein aggregation produced by oxidative stress and protect against cell death	Wilhelmus et al., 2006; Mathys et al., 2019
	Cxcl10	Chemokine	Recruitment of oligodendrocytes and remyelination	Omari et al., 2005
	Cd44	Involved in cell-cell interactions, cell adhesion, and migration	Identify astrocyte-restricted precursor cells	Liu et al., 2004
	Cp	Oxidase	Reducing ICH-induced brain injury	Liu et al., 2019
	Vim	Intermediate filament	Also expressed by endothelial cells, vascular smooth muscle cells, and immature astrocytes	Zamanian et al., 2012
	Gfap	Intermediate filament	Widespread Released by injured astrocytes. Cleavage product found in CSF and plasma	Hol and Pekny, 2015
A1	H2-T23	Major histocompatibility complex		Escartin et al., 2021
	Serpina1	Serine protease Inhibitor	Expression in whole blood links with an increased risk of large artery atherosclerotic stroke	Liu Q. et al., 2020
	H2-D1	Major histocompatibility complex	Altered synapse regulation and impaired synaptic plasticity with aging	Mangold et al., 2017
	Ggta1	Galactosyltransferase	Significant responses activated in the AD model mice exposed to chronic intermittent hypoxia	Macheda et al., 2019
	Fbln5	Promotes adhesion of endothelial cells and play a role in vascular development and remodeling	Antioxidant and antagonize tumor angiogenesis *in vivo*	Guo et al., 2016
	Fkbp5	Play a role in immunoregulation and basic cellular processes involving protein folding and trafficking	Loss of FKBP5 decreased hypothalamus-pituitary-adrenal axis reactivity and glucocorticoid receptor expression changes in response to stressors.	Touma et al., 2011
	Psmb8	Proteasome subunit	Regulates glioma cell migration, proliferation, and apoptosis	Mega et al., 2020
	Srgn	Be associated with the macromolecular complex of granzymes and perforin, which may serve as a mediator of granule-mediated apoptosis.	Enhance glioblastoma growth	Mega et al., 2020
	Amigo2	Adhesion molecule	Involvement in neuroprotection	Laeremans et al., 2013
A2	Clcf1	Potent neurotrophic factor, B-cell stimulatory agent, and neuroendocrine modulator of pituitary corticotroph function	As a neuroimmune-endocrine modulator of the hypothalamus-pituitary-adrenal axis stress response	Auernhammer et al., 2003
	Tgm1	Transglutaminase	Monocyte infiltration was significantly correlated with Tgm1 expression after TBI	Kjell and Götz, 2020
	Sphk1	S1P plays a crucial role in TNF-alpha signaling, and the NF-κB activation pathway is essential in inflammatory, antiapoptotic, and immune processes.	SphK1 activity is stimulated under low oxygen conditions and regulated by reactive oxygen species	Ader et al., 2008
	Cd109	Binds to and negatively regulates signaling by transforming growth factor-beta (TGF-beta).	CD109/STAT3 axis as crucial for the maintenance of stemness and tumorigenicity of glioma stem cells	Filppu et al., 2021
	Ptgs2	Responsible for the prostanoid biosynthesis involved in inflammation and mitogenesis	Inhibit ferroptosis in ICH and exerted a long-term cerebroprotective effect	Chen et al., 2019
	Emp1	Epithelial membrane protein	A novel tight junction protein of the blood-brain barrier	Bangsow et al., 2008
	Tm4sf1	Regulation of cell development, activation, growth, and motility		Zamanian et al., 2012
	Cd14	Mediate the innate immune response to bacterial lipopolysaccharide	Soluble CD14 can also act as a direct agonist for TLR2	Bsibsi et al., 2007

However, it should be realized that the A1/A2 paradigm is a simple and questionable classification method. On the one hand, we do not fully understand the function of A1/A2 astrocytes. Depletion of C3d^+^/GFAP^+^ A1 aggravated cortical degeneration after moderate controlled cortical impact (CCI) (Myer et al., 2006). At the same time, neuron-derived neurotrophic factor (NDNF) released by S100A10^+^/GFAP^+^ A2 can be bound to the P75(NTR) receptor to induce neuronal apoptosis (Lee et al., 2001). On the other hand, RAs may have a more detailed phenotypic classification. In recent years, with the development of single-cell sequencing technology, RAs has shown significant heterogeneity at the transcriptomic level in senility (Boisvert et al., 2018) and many CNS diseases such as Huntington’s Disease, Alzheimer’s disease, and chronic traumatic brain injury (Al-Dalahmah et al., 2020; Pan et al., 2020; Chancellor et al., 2021). Particularly, Hasel’s recent single-cell sequencing of LPS-induced RAs revealed that RAs could be divided into nine subpopulations with distinct gene expression and some subgroups appear in different CNS diseases (Hasel et al., 2021). In the study of ischemic stroke, astrocytes in ischemic penumbra can be divided into seven subgroups and proposed related pathological mechanisms and therapeutic targets (Guo et al., 2021). The spatiotemporal heterogeneity of RAs brings us many questions. First, why do astrocytes show different activation states when receiving the same stimulus at the same time? Second, what triggers turnover between different phenotypes? Finally, is the phenotypic turnover over time due to the transformation of an “old phenotype” into a “new phenotype,” or to the activation of inactive astrocytes into a “new phenotype?”

Therefore, we speculated that RAs might have a more complex model of action in stroke. For example, (1) In the normal brain, astrocytes may have a pre-state of nerve injury or nerve protection. (2) A1 and A2 may be subdivided into more detailed subtypes or there may be new phenotypes beyond A1 and A2. (3) The activation state of RAs may be related to the degree, time, and location of the injury. In order to answer and verify these questions and hypotheses, we need to collect comprehensive multidimensional data of RAs using multiple techniques in the future to establish dynamic models of RAs phenotypes and identify specific functional patterns of different RAs.

## Pathological Role of Reactive Astrocytes

As a neurovascular unit (NVU) component, astrocytes interact with most of the cellular components of the CNS by extending many fine processes. Consequently, astrocytes can serve as bridges between different cells to regulate the transmission of information and substances and to support the CNS functioning normally such as maintenance of internal environment homeostasis, BBB conservation, and neuronal signaling modulation (Simard et al., 2003). After the stroke, a series of cellular and molecular events induced by the breakthrough of blood components into the brain parenchyma or the sudden interruption of blood flow and subsequent reperfusion dramatically alter the astrocyte’s support function. In response to injury, the gene expression level of astrocytes changes observably, and a large number of bioactive molecules are transcribed, translated, assembled, and transported to the membrane or extracellular. These biomolecules not limit the damage scale and promote the removal of necrotic material but also lead to “secondary damage” that aggravates pathological reactions.

### Roles of Reactive Astrocytes in Neuro-Inflammation

After the occurrence of stroke, drastic changes in the internal environment stimulate the injury and the surrounding area cells to release a large number of inflammatory factors (see Table 2), forming an “inflammatory factor storm.” RAs are both the upstream cell and the target cell of inflammatory cytokines and may be involved in the occurrence and elimination of the inflammatory response through various pathways. Our study found that proteinase-activated receptor 4 (PAR4) expressed on the cell membrane of astrocytes detects thrombin released by tissues and activates Tab2/NF-κB signaling pathway to initiate inflammatory injury mechanism (Luo et al., 2021). In addition, RAs produces and releases a large number of inflammatory mediators [such as TNF-α, IL-1α and IFN-γ (Nayak et al., 2012; Doll et al., 2015)] and free radicals [such as NO, superoxide dismutase and peroxynitrite (Gouix et al., 2014)], which directly or indirectly induce neuroinflammation leading to neuronal apoptosis and necrotic death. RAs also secret anti-inflammatory cytokines that inhibit the inflammatory process. Peng et al. (2020) found that DJ-1 (also known as Park7) is highly expressed in astrocytes around the infarct area, and DJ-1 negatively regulates the inflammatory response by promoting the interaction between SHP-1 and TRAF6, thus inducing the dissociation of NLRX1 and TRAF6. Mtlik et al. observed upregulation of mesencephalic astrocyte-derived neurotrophic factor (MANF) and brain dopamine neurotrophic factor (CDNF) in animal stroke models, which can prevent ER stress-induced overactivation and inhibit secretion of pro-inflammatory cytokines IL-1β, TNF-α, and IL-6 (Shen et al., 2012; Cheng et al., 2013; Zhao et al., 2013; Mätlik et al., 2018).

**TABLE 2:** Reactive astrocyte associated inflammatory factors.

Sort	Name	Function	References
Pro-inflammation	TNF-α	Released by mechanically activated astrocytes Promote synaptic damage	Lantoine et al., 2021
	IL1-β	Mice lacking both forms of IL-1 exhibited dramatically reduced ischemic infarct volumes compared with wild type	Boutin et al., 2001; Lau and Yu, 2001
	INF-γ	Directly associated with stroke-induced neurodegeneration	Lau and Yu, 2001; Seifert et al., 2014
	PAR4	Detects thrombin and activates Tab2/NF-κB signaling pathway to initiate inflammatory injury mechanism	Luo et al., 2021
	IL-15	Predisposed microglia to an inflammatory phenotype	Shi et al., 2020
	IL-6	Enhance anti-apoptosis of injured astrocytes and protected neurons against insult	Ali et al., 2000; Lau and Yu, 2001
	VEGF	Increases the permeability of the blood-brain barrier	Min et al., 2017
	MMPs	Basement membrane degradation and BBB integrity destruction	Wiese et al., 2012
Anti-inflammation	TGF- β	Inhibit post-stroke inflammatory response by inhibiting the NF-κB signal of microglia	Cekanaviciute et al., 2014
	DJ-1	Negatively regulates the inflammatory response by promoting the interaction between SHP-1 and TRAF6	Peng et al., 2020
	MANF	Inhibit secretion of pro-inflammatory cytokines IL-1β, TNF-α, and IL-6	Arrieta et al., 2020
	CDNF	Inhibit secretion of pro-inflammatory cytokines IL-1β, TNF-α, and IL-6	Cheng et al., 2013

As a bridge between many cell types, astrocytes’ involvement in regulating inflammatory responses is closely related to their interactions with them and other cell types. In previous studies, for instance, this process was considered inseparable from astrocyte and microglia reciprocal action (Sacristán, 2020). Microglia can initiate inflammatory astrocyte activation. In experimental and human stroke, IL-6, IL-1α, and TNFα are abundantly secreted by microglia, these inflammatory factors promoted the transformation of astrocytes to neuroinflammation phenotype, which lost the ability to promote neuronal survival and growth, synaptogenesis, and phagocytosis as well as induced the death of neurons and oligodendrocytes (Lambertsen et al., 2012; Liddelow et al., 2017). On the other hand, RAs similarly regulate the functional status of microglia. Il-15 expression in astrocytes was significantly up-regulated in intracerebral hemorrhage (ICH) mouse models, and IL-15 predisposed microglia to an inflammatory phenotype (Shi et al., 2020). Transforming growth factor-β (TGF-β) secreted by RAs is one of many cytokines that effectively regulate inflammatory response and inhibit post-stroke inflammatory response by inhibiting the NF-κB signal of microglia (Cekanaviciute et al., 2014).

In the context of inflammation, astrocytes act as regulators or amplifiers of the internal environment of the CNS, constituting a complex and dynamic system of reactions. However, most of the current studies focus on the effect of a certain inflammatory molecule on neuroinflammation, and lack of studies on the comprehensive effect of multiple factors, such as the interference of age and gender on the intensity and type of inflammatory response. In the future, we need to further investigate the influence of multiple factors such as age, gender, area of injury, and current treatment methods on secondary inflammation after the stroke, as well as the role that astrocytes play in this process, in order to find effective ways to alleviate or contain neuroinflammation to improve the prognosis and the quality of life of patients.

### The Bilateral Effect of Reactive Astrocytes in Blood Brain Barrier Disruption and Repair

Within the CNS, the astrocytes endfeet covers almost all capillaries and form an astrocyte boundary membrane outside endothelial cells in the mature brain. The normal morphology and function of the astrocyte boundary membrane are essential in maintaining the integrity of the BBB. knockdown of laminin on astrocyte boundary membrane resulted in decreased expression of tight junction protein in vascular endothelial cells and increased permeability of BBB (Yao et al., 2014). The maintenance of normal BBB function also requires astrocytes regulation. Astrocytes participate in the transport of substances between the CNS and the circulatory system, which prevents the invasion of harmful substances such as circulating antigens (Iadecola and Nedergaard, 2007; Nuriya et al., 2013) and regulate the two-way exchange of water as well as solutes on both sides of the BBB (Fukuda and Badaut, 2012; Shi Z. F. et al., 2021). What is more, astrocytes sense changes in neuronal metabolism and activate their calcium channels, releasing transmitters to regulate blood vessel diameter and blood flow to meet neuronal oxygen and energy needs (Gordon et al., 2007, 2011; Marina et al., 2020; Bonvento and Bolaños, 2021).

**FIGURE 1:**
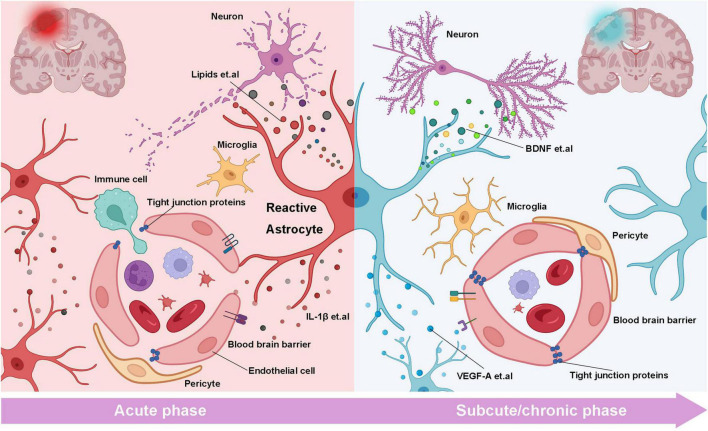
The bilateral roles of reactive astrocytes on the neurons and BBB in the different stages of stroke. In the acute stage of stroke, RAs releases inflammatory factors (such as IL-β) to disrupt tight junction and BBB structure, resulting in the infiltration of immune cells. Meanwhile, saturated fatty acids and inflammatory cytokines from RAs damage synapses and induce neuronal necrosis or apoptosis. In the subacute and chronic phases, RAs release cytokines such as VEGF-A to repair BBB and promote angiogenesis. On the other hand, neurotrophic factors derived from RAs such as BNDF promote axonal regeneration and synaptic reconstruction (created with BioRender.com).

Nevertheless, after the stroke, BBB damage leads to continuous leakage of blood components, which leads astrocytes to activate and convert dramatically. Moreover, the activated astrocytes further aggravate the damage degree of BBB (Figure 1). RAs secrete many inflammatory factors in the acute stage of stroke, which directly or indirectly aggravates the functional damage of BBB. Argaw found that inflammatory mediator IL-1β significantly increased the expression of vascular endothelial growth factor A (VEGF-A) in RAs (Argaw et al., 2012). VEGF-A induces down-regulated claudin-5 and occludin in endothelial cells, which ultimately destroy tight junctions between endothelial cells and significantly increase the extravasation of fibrinogen, albumin, and Ig. In the early ICH, heme and thrombin in plasma enter the brain parenchyma through the broken BBB. Heme activates TLR2 on astrocytes surface (Min et al., 2017), thrombin binds to PAR-1 on astrocytes endfeet (Rajput et al., 2014). Both induce increased expression of matrix metalloproteinases (MMPs) in astrocytes, which are thought to be closely related to basement membrane degradation and BBB integrity destruction (Wiese et al., 2012). Our study found that the adiponectin receptor 1 (APNR1) is expressed explicitly on RAs around capillary after ICH. Specific activation of APNR1 can significantly reduce the damage of the BBB (Wu et al., 2020). In addition, astrocytes respond to changes in osmotic pressure induced by BBB destruction through AQP4. The expression of AQP4 in astrocytes endfeet was significantly increased in the ischemic core and penumbra 1 h after vascular occlusion, and AQP4 spread over the astrocyte membrane surface 48 h later. These two periods coincide with the peak of cerebral edema (Manley et al., 2000). Moreover, AQP4 loss in mice can reduce astrocytes swelling and brain edema and improve neurological recovery after ischemia stroke (Shi X. et al., 2021).

In another aspect, other literature has reported that RAs can promote stroke recovery by repairing BBB or promoting angiogenesis (Figure 1). When BBB injury occurs, the intact astrocyte boundary membrane can partially compensate and restore BBB function. After the stroke, astrocytes up-regulate the expression of claudin 1, Claudin 4, and other tight junction proteins in the boundary membrane formed by astrocytes. Alternatively, the Sonic hedgehog (Shh) release may up-regulate capillary endothelial tight junction protein and initiate compensatory repair, promoting BBB recovery (Tian and Kyriakides, 2009; Wang et al., 2014; Liebner et al., 2018). Whereas, when the physical connection between the astrocyte boundary membrane and vascular endothelial cells is structurally damaged, the impaired BBB function cannot be compensated, leading to the aggravation of the secondary brain injury (Horng et al., 2017). In ICH, RAs upregulates heme oxygenase-1 (HO-1), which can resolve heme that flow into the brain parenchyma. Ho-1 overexpression can significantly improve BBB leakage and neurological dysfunction (Chen-Roetling et al., 2017). RAs can also promote stroke recovery by promoting angiogenesis. VEGF-C and VEGF-A secreted by RAs specifically bind to their receptors FLT-4 and FLT-1, respectively, to promote angiogenesis and contribute to spontaneous reperfusion after ischemic stroke (Gu et al., 2001). Notably, high mobility group protein B1 (HMGB-1) has been implicated in inducing inflammatory immune response after brain injury. Hayakawa found that HMGB-1 released by astrocytes promotes endothelial progenitor cell (EPC) mediated neurovascular remodeling in ischemic stroke models. Inhibition of HMGB1 by siRNA can block the pro-angiogenic response of EPC, decrease angiogenesis in damaged areas, and aggravate neurological deficits (Hayakawa et al., 2012).

The bidirectional function of RAs on BBB may be due to differences in the epigenetics of astrocytes at different stages of the stroke (Figure 1). This requires researchers to further clarify the epigenetic states such as genomic methylation and acetylation of RAs cells at different periods and the mechanisms involved. Secondly, in the normal brain, astrocytes are involved in regulating cerebral blood flow to meet the needs of brain metabolism, but there are still few studies on the changes of astrocytes’ blood flow regulation function in disease conditions. Exploration of it will help us further understand the related mechanisms of vascular remodeling and blood flow recovery after stroke and find emerging therapeutic targets.

### Reactive Astrocytes With Neurotoxicity and Neuroprotection

Astrocytes are regarded as the support cells of neurons, and their dynamic interactions with neurons are crucial to facilitating the formation and stability of synapses and regulating their structure and functions (Nishida and Okabe, 2007). In embryos, contact between embryonic hippocampal neurons and astrocytes activates the neuron’s protein kinase C (PKC) signal, promoting the formation of excitatory synapses (Hama et al., 2004). Astrocytes also phagocytose unnecessary excitatory synaptic connections in the adult hippocampus, critical for supporting cognitive function (Lee et al., 2021). Secondly, astrocytes can sense and stick to synapses, and protrude into the nerve sheath to wrap around synapses. A single cortical astrocyte encloses an average of four neuron bodies and contacts 300–600 neuronal dendrites, forming synaptic “islands” through which astrocytes can synchronize and regulate neural networks (Bushong et al., 2002; Allen, 2014; Yang et al., 2019). Astrocytes can quickly clear neurotransmitters released from synapses and limit their “overflow” to other synapses through physical barriers so that these transmitters cannot interfere with the transmission of other nerve signals (Chen and Swanson, 2003; Dallérac et al., 2018; Todd and Hardingham, 2020).

After the stroke, the supporting function of astrocytes to neurons is significantly changed. In addition to regulating the state of neurons by intervening in the inflammatory response and BBB leakage, there is also evidence that RAs themselves can directly interfere in the survival and repair of injured neurons (Figure 1). The traditional view is that RAs have significant neurotoxicity, and the formation of RAs after stroke is associated with worsening neurological prognosis. Guttenplan demonstrated that the C3d^+^/GFAP^+^ A1 release of saturated lipids activates adipocyte apoptosis-related pathways that directly drive neuronal and oligodendrocyte apoptosis (Guttenplan et al., 2021). RAs release S-100β to enhance the expression of inducible nitric oxide synthase (INOS), leading to NO-mediated neuronal death (Zhao and Rempe, 2010). In addition, RAs interfere with neuronal repair and synaptic reconstruction after the stroke. Scarring of RAs is also thought to be a significant obstacle to axon regeneration in the CNS. The physical barrier formed by the scar prevents the new axons from passing through the damaged area. Hara proved that blocking type I collagen formation to prevent astrocyte scar formation can improve axon regeneration and restore neural function (Hara et al., 2017). New studies have shown that RAs phagocytose newly formed synapses and inhibit the recovery of nerve function after the stroke. Especially in the ischemic stroke model, astrocytes transform into phagocytic phenotypes by upregulation ABCA1 and its pathway-related molecules MEGF10 and GULP1. Phagocytic astrocytes can engulf synaptic components marked by presynaptic and postsynaptic proteins. Inhibition of this pathway can reduce the phagocytosis of astrocytes, improve neurobehavioral outcomes and alleviate brain injury (Schilling et al., 2005; Morizawa et al., 2017; Shi X. et al., 2021).

A new view suggests that injury factors activate downstream pathways of astrocytes and have neuroprotective functions (Figure 1). Ischemic stroke induces the secretion of thrombin from neurons, which binds to PAP1 on the astrocyte surface to induce the production of neuroprotective phenotype astrocytes and block neuronal death induced by oxygen and glucose deprivation (OGD) (Rajput et al., 2020). RAs can also release a variety of neurotrophic factors. RAs highly expressed BDNF, nerve growth factor (NGF), and fibroblast growth factor (FGF) can activate TrkB receptor, TrkA receptor, and FGFR on the surface of neurons, respectively, improve the tolerance of neurons to injury factors and promote neuronal survival (Tokumine et al., 2003; Tejeda et al., 2019). Clinical studies have also confirmed that the release level of the neurotrophic factor is positively correlated with the outcome of stroke patients (Sobrino et al., 2020). RAs are a pivotal participant in regulating synaptic reconstruction after stroke. In focal ischemia model mice, astrocytes significantly increased Thrombospondins 1 and 2 (TSP-1/2) expression and inhibition of TSP-1/2 expression showed decreased synaptic density and axonal sprouting defects (Liauw et al., 2008). Moreover, astrocytes form a network of connections between the nerve germinal center (sublateral ventricle) and the ischemic area and secrete stromal cell-derived factor-1 (SDF-1) that is the chemokine of neuroblasts, guiding neuroblasts migrating to the cerebral infarction area (Thored et al., 2006; Zhang et al., 2007, 2018). To some extent, neuroblasts can differentiate into neurons and repair defective neurons.

In the study of the damage and repair of neurons by RAs in the stroke, we found that many processes are associated with the promotion and reconstruction of neurons by astrocytes during nervous system development. Many related genes are reactivated, transcribed, and expressed by injury factors. Compared to the growth process, the intense stimulation of stroke disrupts the regulatory balance. However, which factors are involved in the reactivation process and the underlying mechanisms still need to be further explored and discovered by future researchers. This not only helps us to further understand the supporting mechanism of astrocytes for neurons but also helps us to find ways to reconstruct the balance of astrocyte regulation after the stroke.

## Advances in Tools for Astrocyte Analysis

Over the past few years, several complementary techniques and tools have encouraged progress in exploring astrocytes’ molecular mechanisms, signaling pathways, and functional patterns (Yu et al., 2020). First of all, in combination with a specific mouse lineage and anti-astrocyte specific antibodies, astrocytes can be specifically isolated and purified by FACS, immunostaining, and activated magnetic cell sorting to improve the accuracy and efficiency of single-cell transcription sequencing (Orre et al., 2014; Holt and Olsen, 2016; Zhang et al., 2016; Holt et al., 2019). Secondly, the latest technology has enabled direct analysis of mRNA transcribed in specific populations of cells in the brain without isolating cells. A technique called translational ribosomal affinity purification (TRAP) is the gene-targeted expression of EGFP-labeled ribosomal protein L10a, which can be obtained using EGFP-specific antibodies, enabling sequencing of associated ribosomal mRNA (Doyle et al., 2008). RiboTag is a related method (Sanz et al., 2009). Currently, RiboTag can be deployed in astrocyte around the lesion by astrocyte-specific RiboTag AAV vector to analyze the changes in astrocyte transcription levels after the onset of disease (Yu et al., 2018; Nagai et al., 2019). Finally, the latest visualization techniques can provide a more intuitive view of astrocyte ultrastructure. Super-resolution microscopy, combined with mathematical reconstruction to improve spatial resolution below the refractive index limit of the optical microscope, is capable of imaging the tiny projections of astrocytes and certain synaptic proteins within them. This approach is able to visualize astrocyte structure at tens of nanometers, which has excellent potential for observing astrocyte synaptic changes after stroke (Heller et al., 2020). The application of new technologies and tools in the CNS promises to research and raises further scientific questions to improve researchers’ understanding of astrocyte function after stroke.

## Therapeutic Potential of Targeting Astrocytes

For decades, stroke treatment has focused on alleviating irreversible neuronal damage, but therapies have failed to provide significant clinical benefits. In recent years, the NVU composed of neurons, glial cells, and vascular systems has gradually been regarded as an essential factor affecting various neurological diseases, including stroke (Guo and Lo, 2009). Therefore, future therapies should expand the scope of neuroprotection to cover the entire NVU. Astrocytes, as a large cell population in contact with all components of NVU, provide broad prospects for clinical stroke treatment (see Table 3).

**TABLE 3:** Stroke clinical studies of drugs for reactive astrocyte.

Drug	Function	Level	Outcome	References
EPO	Inhibit astrocyte swelling in the penumbra through an effect on AQP4 water permeability	Prospective, randomized, placebo-controlled trial	Significantly improved long-term neurological outcomes in patients after ischemic stroke.	Tsai et al., 2015
Selegiline	Promote astrocytes to secrete the neurotrophic factor	Randomized, double-blind, and placebo-controlled phase II study	Seems to be beneficial after a cerebral infarction, although they did not reach statistical significance.	Sivenius et al., 2001
Edaravone	Reduced astrocyte damage markers	Multicenter, randomized, double-blind, multiple-dose, active-controlled, phase II clinical trial	Safe and well-tolerated at all doses, although no significant improvement in functional outcomes was observed at 90days	Uno et al., 2005
Arundic Acid (ONO-2506)	Exerts neuroprotective effects through inhibition of astrocytic synthesis of S100B	Multicenter, dose-escalating, randomized, double-blind Phase I trial	A dose of 8 mg/kg/h AA produced a favorable trend in reduction of the National Institutes of Health Stroke Scale	Pettigrew et al., 2006
Fingolimod (FTY720)	Down-modulates S1P1 in astrocytes to reduce astrogliosis and help restore gap-junctional communication of astrocytes with neurons and cells of the blood-brain barrier.	2-arm, evaluator-blinded study	In patients with small- to moderate-sized deep primary supratentorial ICH, administration of oral fingolimod within 72 h of disease onset was safe, reduced PHE, attenuated neurologic deficits, and promoted recovery.	Fu et al., 2014
Glyburide	Target Sur1-transient receptor potential melastatin 4, which co-assembles with aquaporin-4 to mediate cellular swelling of astrocytes	Double-blind, randomized, placebo-controlled phase 2 trial	Intravenous glyburide was well tolerated in patients with large hemispheric stroke at risk for cerebral edema.	Sheth et al., 2016
Cilostazol	Limit ischemia-reperfusion injury with advanced glycation endproducts by improving the tight junction proteins and inhibiting TGF-β1 signaling	Multicenter, open-label, randomized controlled trial	The combination of cilostazol with aspirin or clopidogrel had a reduced incidence of ischemic stroke recurrence and a similar risk of severe or life-threatening bleeding compared with treatment with aspirin or clopidogrel alone in patients at high risk for recurrent ischemic stroke.	Toyoda et al., 2019

### Astrocyte Phenotype Modulations

As mentioned above, different phenotypes of astrocytes have their functional characteristics. Can we promote stroke recovery by modulating the phenotype of RAs toward neuroprotective type? *In vitro* and animal models, related interventions have been found to inhibit the inflammatory response, reduce BBB damage, alleviate injury and surrounding edema volume, and improve neurological function. For example, Liu M. et al. (2020) found that cottonseed oil (CSO) can inhibit the C3d^+^/GFAP^+^ astrocytes by blocking the TLR4/NF-κB pathway and reducing the infarct volume and inflammatory response in the middle cerebral artery occlusion (MCAO) rat model. The glucagon-like peptide-1 receptor (GLP-1R) agonist Exendin4 (EX-4) binds to astrocyte surface GLP-1R to protect the BBB and reduce secondary inflammation caused by cerebral ischemia. Shan demonstrated that EX-4 improved neurological deficit scores in the rat model of MCAO, reduced infarct size, and reduced levels of VEGF-A, MMP-9, CXCL-1, and MCP-1 derived from astrocytes (Shan et al., 2019). Our study found that classical antioxidant and anti-inflammatory adiponectin mimicking peptides can be enriched on the surface of the astrocyte membrane around BBB and inhibit the inflammatory damage of RAs on BBB and neurons by activating AMPK-DRP1 signaling (Wu et al., 2020). In the study of RAs phenotype, numerous intervention sites have been exposed, and the intervention effects of each site may be unrelated to or affect each other, which undoubtedly brings difficulties for researchers and clinicians to choose. In future translational clinical studies, researchers need to carefully identify the interrelationships and effects of each point to develop the most effective comprehensive intervention measures.

### Astrocyte Scar

Traditionally, astrocyte scarring is the main reason for the failure of axon regeneration in severe central nerve injury. Inhibition of scar formation can improve axonal regeneration and nerve function. Previous studies have illustrated that the main mechanism of inhibition of axon regeneration by astrocyte scar is the production of CSPG (Silver and Miller, 2004). However, the up-regulated CSPG4 and CSPG5 in astrocyte scar can also promote axon growth, and only when the scar formed by astrocytes is present, the related genes of axon growth will be activated (Anderson et al., 2016). The specific relationship between glial scar and axon regeneration as well as the prognosis may be related to the injury site, duration of the stroke, and the response degree of astrocytes. Previous therapies that inhibit scar formation by inhibiting astrocyte reactivity have not achieved the desired results. Therefore, the opposite strategy has been proposed to enhance RAs’ reactivity and promote their neuroprotective function. The monoamine oxidase B inhibitor selegiline can strengthen the reaction of astrocytes to injury and increase the expression of NGF, which has been proved to alleviate ischemic injury of cortical tissues after MCAO in mice and improve the prognosis of patients (Bartolo et al., 2015; Nardai et al., 2015). Whether inhibition of astrocyte scar formation can improve the prognosis of stroke patients still requires further exploration by researchers.

Given that scars are indeed a physical barrier to axon regeneration, we hypothesized that the best strategy might be to exert the protective function of astrocytes in the early stage and select appropriate intervention sites to inhibit scar formation in the middle or late stages. For example, ephrin-A5 expressed by RAs in the peri-infarction region can inhibit axon regeneration, and inhibition of ephrin-A5 in the peri-infarction area can promote axon growth on day seven after stroke (Overman et al., 2012), which provides a long treatment time window so that has broad clinical prospects.

### Astrocyte in Stem Cell Therapy

In recent years, stem cell therapy using embryonic stem cells or induced pluripotent stem cells to generate neurons has been considered to fill in the defective neurons and restore normal brain function in the damaged area (Yu et al., 2019; Sun et al., 2020; Asgari Taei et al., 2021). However, this cell transplantation method faces risks such as immune rejection, tumor formation, and differentiation uncertainty (Lee et al., 2013; Lukovic et al., 2014). Recent studies have discovered that astrocytes provide a new possibility for stem cells in stroke treatment. Astrocytes can induce stem cell proliferation and differentiation. In the co-culture of human umbilical cord mesenchymal stem cells (huc-Mscs) with LPS-induced RAs, it was found that huc-Mscs showed a higher S phase ratio, and BDNF secreted by astrocytes could induce the neural differentiation of exogenous huc-Mscs (Shi et al., 2016). In addition, astrocytes may have the potential to transform directly into neurons to repair damaged areas. Neural stem cells can migrate from the sub-ventricular zone (SVZ) region to the site of injury, where they differentiate into RAs subsets, and these SVZ-derived RAs can be transformed into neurons *in vivo* through forced expression of Ascl1 (Doetsch et al., 1999). Meanwhile, astrocytes can also reverse into radial glial stem cell rNSC and then differentiate into neurons (Niu et al., 2015). Surprisingly, neuroD1 can promote the direct transformation of astrocytes into functional neurons (Zamboni et al., 2020), and these neurons produced by astrocytes have a favorable functional state such as can participate in the establishment of synaptic networks and electrical signal transmission (Berninger et al., 2007; Zhang et al., 2015). However, the transformation of astrocytes into neurons is still controversial. Wang et al. (2021) found that overexpression of NeuroD1 does not change the fate of astrocytes according to cell lineage tracing, but NeuroD1 affects the specificity of the GFAP promoter, which is ultimately expressed in neurons. Nevertheless, the role of astrocytes on stem cells and their dryness is still a significant research area in the field of neural repair in the future.

### Chinese Medicines

Chinese herbal medicines (CHM) have a long tradition in treating stroke and related disorders. Multiple *in vitro* and *in vivo* studies have shown that many CHM can promote angiogenesis, inhibit inflammation and protect neural function through astrocytes (see Table 4). Ginkgo Diterpene Lactones (GDL) are extracts of active components from Ginkgoaceae leaves. GDL can inhibit astrocyte activation in rat middle cerebral artery occlusion/reperfusion (MCAO/R) and neuronal oxygen-glucose deprivation/reoxygenation (OGD/R) models and inhibit inflammation by inhibition of the TLR4/NF-κB pathway (Li et al., 2020). Ginsenosides, the bioactive components of Panax ginseng, can improve the mitochondrial oxidative phosphorylation efficiency and reduce ROS production of astrocytes in the OGD/R model, thereby protecting astrocytes (Xu et al., 2019). 1,3,7-trihydroxyxanthone is a compound isolated from Polygalae Radix, which can significantly stimulate high expression of neurotrophic factors (NGF, BDNF, etc.) in astrocytes and promote neuronal survival (Yang et al., 2018). Clinical and large-scale population epidemiological studies have proved the potential benefits of Chinese herbs and herbal formulations in the treatment of ischemic stroke (Chen et al., 2012; Hayat et al., 2015). CHM will have a broad application prospect in treating stroke in the future.

**TABLE 4:** Chinese herbal medicines.

Active components	CHM	Function	References
GDL	Ginkgoaceae	Inhibit astrocyte activation in rat (MCAO/R) and neuronal (OGD/R) models and inhibit inflammation by inhibition of the TLR4/NF-κB pathway	Li et al., 2020
Ginsenosides	Panax ginseng	Protect astrocytes by improving the mitochondrial oxidative phosphorylation efficiency and reduce ROS production of astrocytes in the OGD/R model	Xu et al., 2019
1,3, 7-trihydroxyxanthone	Polygalae Radix	Stimulate expression of neurotrophic factors (NGF, BDNF, etc.) in astrocytes and promote neuronal survival	Yang et al., 2018
Triptolide	Tripterygium wilfordii Hook F	Increased NGF mRNA expression and intracellular NGF and NGF levels in astrocytes	Xue et al., 2007
D. moldavica L.	Dracocephalum moldavica L.	It protects astrocytes from oxidative stress by inhibiting mitochondrial dependent pathways associated with CaMKII/P38MAPK/ERK1/2 and PI3K/AKT/mTOR pathways	Zheng et al., 2020
Salvianolate lyophilized injection	Danshen	It inhibited the activation of astrocytes, significantly reduced the neurological deficit score and infarct volume, and increased regional cerebral blood flow.	Wang et al., 2018
Xueshuantong	Sanqi	It inhibited the activation of astrocytes, significantly reduced the neurological deficit score and infarct volume, and increased regional cerebral blood flow.	Wang et al., 2018

### Therapeutic Limitation

Unfortunately, all of these interventions have been unable to show the desired results in clinical trials. Above all, effective drug concentration may not be achieved in the injured area because related drugs are difficult to cross the BBB. Excitingly, a promising technology holds out the possibility of breaching the BBB’s limits. The ability of platelet membranes to cross barriers and target damage areas is transferred to microglia by membrane fusion. Ultrasound responsive IL-4 loaded liposomes were then attached to engineered microglia *via* bioorthogonal reaction to achieve remote directional control of specific anti-inflammatory phenotypic polarization of *in situ* microglia (Li et al., 2021). The broad potential of its ability to span BBB and target drug release deserves further investigation: (1) Whether this platform can be used to regulate RAs phenotypes after stroke; (2) Whether similar methods can be used to transplant astrocytes with neuroprotective phenotypes into the injured area by injection.

In the second place, as mentioned above, RAs have complex subsets and a certain temporal variation trend in the area of stroke injury. only at a single point in time for a single pathological factor intervention may be challenging to achieve satisfactory results or even counterproductive. This will require future researchers to further clarify: (1) Whether there are “threshold genes” for the phenotypic transformation of RAs, the phenotypic regulation of RAs can be achieved by manipulating them alone; (2) Whether a multi-pathway combined regulation scheme similar to “cocktail therapy” can be constructed to comprehensively regulate astrocyte function. The resolution of these problems will provide a broad clinical potential for RAs to promote the outcome of stroke.

## Conclusion

Astrocytes are the most abundant glial cells in the CNS. They contact and communicate with other CNS components and participate in normal physiological and pathological reactions of the nervous system structurally and functionally. One of the most significant pathologic features of stroke is RAs formation induced by environmental changes in the CNS. The phenotypic and functional characteristics of RAs differed in different modes and periods of injury. They both repair damaged nerves and inhibit axon regeneration. They also play a protective role in the BBB and increase BBB leakage. Future studies should comprehensively consider the phenotypic transformation of RAs and their bidirectional effects, from which to explore multi-temporal and multi-dimensional intervention methods to ultimately achieve comprehensive treatment to reduce injury and promote repair after stroke. these may be the key to stroke treatment and improving the prognosis of patients.

## Author Contributions

All authors listed have made a substantial, direct, and intellectual contribution to the work, and approved it for publication.

## Conflict of Interest

The authors declare that the research was conducted in the absence of any commercial or financial relationships that could be construed as a potential conflict of interest.

## Publisher’s Note

All claims expressed in this article are solely those of the authors and do not necessarily represent those of their affiliated organizations, or those of the publisher, the editors and the reviewers. Any product that may be evaluated in this article, or claim that may be made by its manufacturer, is not guaranteed or endorsed by the publisher.
